# A phase 1 study of intravenously administered TR-701 FA in subjects with advanced renal impairment

**DOI:** 10.1186/cc12641

**Published:** 2013-06-19

**Authors:** S Flanagan, D Morris, T Boyea, H Dreskin, SL Minassian, H Alcorn, T Marbury, M Abdelhameed, E Fang, P Prokocimer

**Affiliations:** 1 Trius Therapeutics, San Diego, CA, USA; 2 Covance, Madison, WI, USA; 3 DaVita, Minneapolis, MN, USA; 4 Orlando Clinical Research Center, Orlando, FL, USA

## Introduction

The objective of the study was to characterize the safety of tedizolid phosphate (TR-701 FA) and the pharmacokinetics of tedizolid (TR-700), its microbiologically active moiety, in subjects with advanced renal impairment (eGFR <30 ml/minute/1.73 m^2^, not on dialysis) compared with matched subjects with normal renal function (eGFR ≥80.0 ml/minute/1.73 m^2^).

## Methods

Eight subjects with advanced renal impairment and eight matched controls (by age, gender, and body mass index) received a single intravenous infusion of 200 mg TR-701 FA. Serial plasma PK samples were collected from pre-dose through 72 hours post-dose. Plasma samples were analyzed for TR-700 and the following pharmacokinetic parameters were calculated: C_max_, t_max_, AUC_0−∞_, AUC_0−t _λ_z_, CL_sys_, and plasma ${\text{t}}_{1\text{/}2}$.

## Results

Baseline eGFR ranged from 7 to 28 ml/minute/1.73 m^2 ^(including three subjects with eGFR <15 ml/minute/1.73 m^2^). The pharmacokinetics of TR-700 was essentially unchanged in subjects with advanced renal impairment relative to a matched control group. Approximately 8% lower AUC and nearly identical C_max _values were observed in renal impaired subjects relative to matched controls, and other pharmacokinetic parameters were also similar between groups. See Table 1.

**Table 1:** Pharmacokinetic parameters of tedizolid in control and renal impaired subjects

	Geometric mean (GM)			90% Cl	
		
TR-700	Advanced renal impairment	Control	GM ratio	Lower	Upper
AUC_0-t _(μg·hour/ml)	28.4	30.7	0.93	0.70	1.23
AUC_0-∞ _(μg·hour/ml)	28.7	31.0	0.92	0.70	1.23
C_max _(μg/ml)	3.01	3.02	0.99	0.78	1.27

## Conclusion

The TR-700 plasma pharmacokinetic results from this study provide support that no dose adjustment is needed in subjects with advanced renal impairment.

